# Nutritional Quality, Phenolic Compounds, Fatty Acid Composition, Texture, and Sensory Properties of Full‐Fat/Semi‐Fat Quinoa, Chia, or Chickpea Biscuits Developed for Celiac Patients

**DOI:** 10.1002/fsn3.72399

**Published:** 2026-09-23

**Authors:** Seda Yalcin, Azize Atik, İlker Atik

**Affiliations:** ^1^ Food Processing Department, Afyon Vocational School Afyon Kocatepe University Afyonkarahisar Türkiye

**Keywords:** biscuit, celiac, chia, chickpea, quinoa

## Abstract

The objective of this study is to determine nutritional quality, phenolic compounds, fatty acid composition, spread ratio, color, pH, hardness, sensory profile, and energy value of full‐fat and semi‐fat gluten‐free biscuits made with white quinoa, chia seed, or chickpea flours, compared to wheat biscuits. Semi‐fat biscuits were significantly harder than full‐fat biscuits. Gluten‐free biscuits with different fat content had significantly higher levels of phosphorus, magnesium, potassium, caffeic acid, and linoleic acid than wheat biscuits (*p* < 0.05). The protein content of chia (14.04%) and chickpea (12.96%) biscuits was significantly higher than that of quinoa (10.00%) and wheat (8.40%) biscuits (*p* < 0.05). Quinoa biscuits had a significantly higher level of naringin than chia, chickpea, and wheat biscuits (*p* < 0.05). Chia biscuits had the highest levels of total dietary fiber (17.52%), vanillic acid (8.26 mg/kg), rosmarinic acid (630.06 mg/kg), and linolenic acid (39.81%), a type of essential polyunsaturated fatty acid, among biscuits. Chickpea biscuits had a significantly higher level of caffeic acid than quinoa, chia, and wheat biscuits (*p* < 0.05). The spread ratio of gluten‐free biscuits was significantly higher than that of wheat biscuits (*p* < 0.05). Quinoa and chickpea biscuits received sensory scores close to those of the wheat biscuit. Chickpea biscuits had the lowest energy. The results showed that using white quinoa, chia seed, and chickpea flours in biscuits positively affects the nutritional quality, bioactive components, and linoleic acid content. Quinoa, chia and chickpea biscuits can be preferred by celiac patients. Semi‐fat chickpea biscuit can be made as a dietetic product due to having low energy (362.8 kcal/100 g). Gluten‐free biscuits might positively influence children's development because of their high protein, mineral, and linoleic acid content.

## Graphical abstract

This study aims to develop and compare full‐fat/semi‐fat gluten‐free biscuits produced from white quinoa, chia seed, and chickpea flours with wheat biscuits intended for celiac patients. Gluten‐free biscuits had higher levels of phosphorus, magnesium, potassium, caffeic acid, and linoleic acid than wheat biscuits. The spread ratio of gluten‐free biscuits was higher than that of wheat biscuits. Semi‐fat biscuits give lower energy than full‐fat biscuits. Gluten‐free biscuits with health‐promoting effects can be made from white quinoa, chia seed, and chickpea.

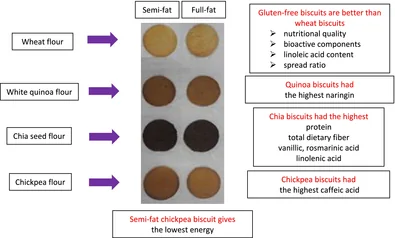

## 1 Introduction

Gluten‐related diseases are classified as autoimmune (celiac disease), allergic (wheat allergy), and nonautoimmune, nonallergic (nonceliac gluten) sensitivity according to their pathogenesis (Kotze et al. 2026). Celiac disease is an immune‐mediated enteropathy caused by gluten exposure in susceptible patients. Improved screening tools showed celiac disease to be a major public health issue with a rapidly increasing prevalence at approximately 1% of the general population (Kurppa et al. 2024). A 20%–30% of autoimmune diseases has been reported in nonceliac wheat sensitivity (Seidita et al. 2025). Clinical studies related to nonceliac wheat sensitivity in the last 5 years are demonstrated to shed light on this complex condition (Manza et al. 2025). Regardless of the presence of symptoms, all patients must follow a gluten‐free diet. The demand for gluten‐free products has increased in the last decade (Shevkani et al. 2022). Gluten‐free products may be associated with decreased quality of life in celiac patients (Machado 2023). There are various studies on replacing wheat with grains, pseudocereals, and legumes such as rice, corn, amaranth, buckwheat, quinoa, millet, beans, chickpeas, and lentils. Gluten‐free products are generally higher in sugar, fat, and salt, while lower in protein and fiber content (Melini and Melini 2019). Gluten‐free products cause nutritional deficiencies (Aljada et al. 2021). Gluten‐free baked products like biscuits often lack nutritional quality due to the limited protein and fiber content of cereal flours like maize and rice (Allouch Tounsi et al. 2025). Gluten‐free products are characterized by lower sensory quality than gluten‐containing products (Drabińska et al. 2016). It is challenging for celiac patients to find suitable food options. Therefore, they prefer packaged gluten‐free products such as biscuits. The biological value of the proteins found in biscuits is important (Di Cairano et al. 2018). In recent years, scientists' efforts to produce biscuits with improved nutritional values and good scores have increased. Allouch Tounsi et al. (2025) reported that legume flour can be used to produce cereal‐based gluten‐free biscuits with improved physical, sensory, and nutritional qualities. Tazoho et al. (2026) demonstrated that gluten‐free biscuits based on tiger nut, dana, avocado, and margarine flours showed good nutritional properties and a low glycemic index for people at risk of diabetes.

Quinoa is a pseudo‐cereal food and is gaining popularity among health‐conscious clientele. It exhibits excellent nutritional quality, being high in protein content with an abundance of essential amino acids, vitamins, and minerals (Chandra et al. 2018; Nisar et al. 2018). It contains 16%–18% protein, with more than 37% of the protein comprising essential amino acids (Drzewiecki et al. 2003), 4.4%–8.8% fat, with the essential fatty acids (linoleic and linolenic acids) accounting for 55%–63% of the total fatty acids, 13.4% total dietary fiber (Alvarez‐Jubete et al. 2010). There are three quinoa varieties. Each quinoa variety has differences in their nutritional profiles, their relevance in food development and their contribution to human health. White quinoa is valued for its versatility and neutral flavor, making it a common choice in food product development. Red quinoa is characterized by its higher antioxidant content, contribute to reducing the risk of chronic diseases and mitigating oxidative stress. Black quinoa contains high nutrients and bioactive compounds, conferring additional health benefits. The utilization of quinoa varieties in product may promote overall health and support disease prevention, reinforcing quinoa's role as a functional food (Pérez‐Viveros et al. 2026). Brito et al. (2015) reported that quinoa flour and flakes positively affected the hardness of gluten‐free biscuits, including maize starch. As the amount of quinoa flour and flakes increased, the lightness decreased, while protein, sugar, and phenolic compounds increased in biscuits.

Chia seeds contain high amounts of protein, dietary fiber, essential fatty acids, antioxidants, vitamins, minerals, and carotenoids (Ayerza and Coates 2011; Reyes‐Caudillo et al. 2008). The phenolic compounds of chia seeds are chlorogenic acid, ferulic acid, caffeic acid, *p*‐coumaric acid, rutin, and quercetin (Laçin and Başman 2025). These components have health benefits for chronic diseases such as obesity, cardiovascular disease, diabetes, and cancer (Ixtaina et al. 2008). Tüter et al. (2025) observed that chia seed increased the total phenolic content of gluten‐free biscuits.

Chickpeas, which are considered a valuable, healthful, and functional food, are an ideal ingredient for improving the nutritional value of biscuits and bakery products due to their high protein, fiber, vitamin, and mineral content (Hefnawy et al. 2012). Chickpeas help regulate blood glucose levels in patients with diabetes by modulating glycemic responses (Hawkins and Johnson 2005). Abd Rabou (2017) found that chickpea flour increased the spread ratio of gluten‐free biscuits made from rice flour. Benkadri et al. (2018) used xanthan gum in rice‐chickpea biscuits and reported that xanthan gum caused increases in the thickness of biscuits.

The present study aims to develop and compare full‐fat and semi‐fat gluten‐free biscuits produced from white quinoa, chia seed, and chickpea flours with wheat biscuits intended for celiac patients. Accordingly, several analyses were performed to compare the nutritional quality, phenolic compounds, fatty acid composition, pH, spread ratio, hardness, color, sensory properties, and energy value of the gluten‐free and wheat biscuits. Furthermore, the impact of fat reduction on biscuit quality parameters was evaluated, aiming to develop new product options for celiac patients, obese individuals, and children. In this study, white quinoa, chia seed, and chickpea flours were used in the production of gluten‐free biscuits to obtain a nutritionally improved and organoleptically acceptable result. There are a few studies on gluten‐free biscuits made with white quinoa and chickpea flours. The primary materials used in these studies are corn starch or rice flour. The difference between our study and other studies is that the main ingredients of our gluten‐free biscuits are only white quinoa flour, chia seed flour, or chickpea flour. There is no other type of flour in the formulation. Although chia seed was examined, its flour has not been used in any study about gluten‐free biscuits to date. It is believed that this study will contribute to the research on gluten‐free biscuits.

## 2 Materials and Methods

### 2.1 Chemicals

Methanol, ethanol, hexane, *n*‐heptane, acetone, hydrochloric acid, nitric acid, hydrogen peroxide, ethyl ester, petroleum ester, sodium phosphate, sodium hydroxide, sodium chloride, formic acid, and boron trifluoride reagent were purchased from Merck (Darmstadt, Germany). Phenolic standards (ferulic acid, caffeic acid, *p*‐coumaric acid, 4‐hydroxybenzoic acid, vanillic acid, naringin, and quercetin) and fatty acid methyl ester mix were purchased from Sigma‐Aldrich (St. Louis, Missouri, USA). Enzymes were purchased from Megazyme International Ltd. (Ireland).

### 2.2 Materials

Soft wheat flour, white quinoa, chia seeds, chickpea flour, sucrose, brown sugar, vegetable fat, sodium bicarbonate, salt, and skimmed milk were purchased from a local market in Türkiye.

### 2.3 Biscuit Production

**FIGURE 1:**
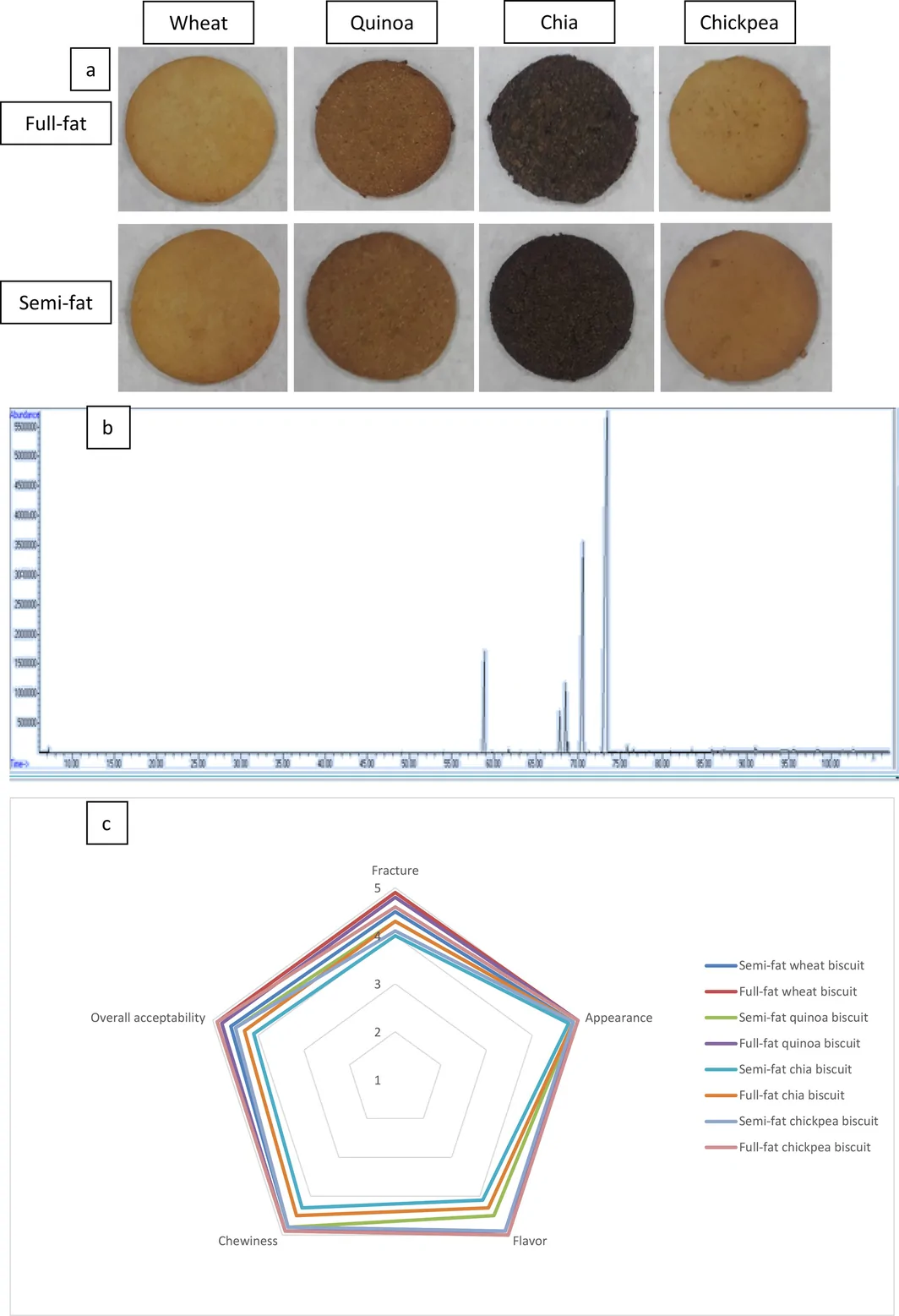
(a) Photographs of biscuits (Wheat; Semi‐fat and full‐fat wheat biscuits, Quinoa; Semi‐fat and full‐fat quinoa biscuits, Chia; Semi‐fat and full‐fat chia biscuits, Chickpea; Semi‐fat and full‐fat chickpea biscuits), (b) GC–MS chromatogram of biscuit, and (c) sensory profile of biscuits.

White quinoa and chia seeds were milled in a grinder (Bosch, Berlin, Germany) and sieved through a 212 μm. Full‐fat (40% fat, flour basis) and semi‐fat (20% fat, flour basis) biscuits were produced from wheat, white quinoa, chia seeds, and chickpea flours according to the method reported by Yalcin et al. (2023). Formulation of biscuits is given in Table 1. Wheat biscuit was produced from wheat flour. Quinoa biscuit was produced from white quinoa flour. Chia biscuit was produced from chia seed flour. Chickpea biscuit was produced from chickpea flour. Photographs of biscuits are shown in Figure 1a.

**TABLE 1:** Formulation of biscuits.

Ingredient (g)	Semi‐fat biscuit	Full‐fat biscuit
Sucrose	25.6	25.6
Brown sugar	8	8
Vegetable fat	16	32
Skimmed milk	18	18
Sodium bicarbonate	1.2	1.2
Salt	0.4	0.4
Flour	80	80

### 2.4 Proximate Composition of Raw Materials and Biscuits

Moisture, protein, fat, and ash contents were determined according to AACCI method 44–01.01 (2010), the method reported by Shea and Watts (1939), the TS EN ISO 11085 method (2015), and the AACCI method 08–01.01 (2010), respectively. The total carbohydrate content was calculated according to the equation reported by Divyashree et al. (2016). The total dietary fiber content was determined according to the AOAC 985.29 and AOAC 991.43 methods (1992). Analyses were done in duplicate.

### 2.5 Mineral Content of Biscuits

Biscuits (1 g) were mixed with 30% H_2_O_2_ (2 mL) and 65% HNO_3_ (8 mL) and heated to 110°C for 15 min, and then microwaved (15 min). The biscuits' phosphorus, magnesium, calcium, and potassium contents were determined according to the method reported by Gopalani et al. (2007). Analyses were done in duplicate.

### 2.6 Energy, Physical, and Color Properties of Biscuits

A biscuit's energy value was determined by its macronutrient content, including carbohydrates, proteins, and fats. It was calculated using Equation (1).

(1) $$\text{Energy}\;\left(\text{kcal}/100\;g\right)=\left(\left(\text{carbohydrate}\times 4\right)+\left(\text{protein}\times 4\right)+\left(\mathrm{fat}\times 9\right)\right)$$

The diameter and thickness of the biscuits were determined using a caliper. The spread ratio of biscuits was calculated as the diameter divided by the thickness. Analyses were done in duplicate.

The color of the biscuits was measured using a Chroma meter (Konica Minolta Cr‐400/410, Japan). Analysis was done in duplicate. Color was expressed as L* (lightness), a* (redness‐greenness), and b* (yellowness‐blueness).

Chroma (C*) and Hue (h*) values of the biscuits were calculated using Equations (2) and (3) reported by Laguna et al. (2011). The total color difference (△E*) of gluten‐free biscuits in comparison to the control biscuits was calculated according to Equation (4), where the suffix denotes the control sample (wheat biscuits) reported by Lu et al. (2022).

(2) $$C^{*}=\sqrt{a^{*2}+b^{*2}}$$

(3) $$h^{*}=\arctan\frac{b^{*}}{a^{*}}$$

(4) $$∆E^{*}=\sqrt{{\left(L^{*}-{L^{*}}_{0}\right)}^{2}+{\left(a^{*}-{a^{*}}_{0}\right)}^{2}+{\left(b^{*}-{b^{*}}_{0}\right)}^{2}}$$

Browning index (BI) was calculated according to the equation reported by Dadalı et al. (2007).

### 2.7 pH of Biscuits

pH measurement of the biscuits was performed according to the method reported by Žilić et al. (2016). Analysis was done in duplicate.

### 2.8 Texture Analysis of Biscuits

The hardness of the biscuits was evaluated using the Texture Analyzer TA.XT. Plus (Stable Micro Systems, Godalming, England), including HDP/90 working platform and HDP/3 PB breaking equipment with a 50 kg load cell and a probe with a pretest speed of 2.00 mm/s, test speed of 0.5 mm/s, posttest speed of 10 mm/s, and trigger force of 5 g. The hardness result was expressed as g force. Analysis was done in duplicate.

### 2.9 Phenolic Compounds of Biscuits

Phenolic compounds of biscuits were analyzed according to the method reported by Yalcin et al. (2023). The peaks were identified by comparing the retention times with phenolic compound standards (vanillic acid, caffeic acid, *p*‐coumaric acid, 4‐hydroxybenzoic acid, ferulic acid, naringin, and quercetin). Quantitative analysis of phenolic compounds was performed by using calibration curves of standards. Phenolic compounds were expressed as mg *per* kg. Analysis was done in duplicate.

### 2.10 Fatty Acid Composition of Biscuits

Biscuits were extracted and methylated according to the AOAC 996.01 method (1992). Fatty acid methyl esters of the biscuits were analyzed according to the method reported by Yalcin et al. (2023). Identification of individual components was performed using a reference fatty acid methyl ester (FAME) mixture (SUPELCO). Analysis was done in duplicate. Fatty acid composition was expressed as g *per* 100 g of individual fatty acids.

### 2.11 Sensory Analysis of Biscuits

Sensory analysis of biscuits was conducted in the food laboratory on a white tile counter according to Ahmed et al. (2014) with some modification. Biscuits were coded numerically. The sensory panel includes 15 trained panelists (students and university academic staff at the Food Processing Department). One of them is a celiac patient. The five‐point scale hedonic test ranged from 1 (disliked at all) to 5 (liked very much). The biscuits were evaluated for fracture, appearance, flavor, chewiness, and overall acceptability.

### 2.12 Statistical Analysis

Statistical differences between biscuits were evaluated using a two‐way analysis of variance (ANOVA) followed by the Duncan test. The main factors are flour type and fat content. Flour types are wheat flour, white quinoa flour, chia seed flour, and chickpea flour. Fat contents are full‐fat and semi‐fat. The difference between groups was significant at *p* < 0.05. All data were analyzed using IBM Statistics SPSS 24.

## 3 Results and Discussion

### 3.1 Nutritional Components of Raw Materials

Moisture, protein, fat, total carbohydrate, total dietary fiber, and ash contents of wheat, white quinoa, chia seed, and chickpea flours are presented in Table 2. All gluten‐free raw materials had significantly higher protein, fat, total dietary fiber, and ash contents, and lower total carbohydrate than wheat flour (*p* < 0.05). Among the gluten‐free raw materials, chia seed flour had the highest protein, fat, total dietary fiber, and ash contents, and the lowest total carbohydrate content.

**TABLE 2:** Nutritional contents of wheat, white quinoa, chia seed, and chickpea flours.

Samples	Moisture (%)	Protein (%)	Fat (%)	Total carbohydrate (%)	Total dietary fiber (%)	Ash (%)
Wheat flour	14.19 ± 0.39b	10.50 ± 0.41d	0.90 ± 0.03d	72.80 ± 1.91a	2.50 ± 0.03d	1.61 ± 0.09c
White quinoa flour	11.40 ± 0.19c	14.50 ± 0.31c	6.80 ± 0.13b	64.40 ± 1.83b	7.40 ± 0.09c	2.90 ± 0.15b
Chia seed flour	5.40 ± 0.13d	22.00 ± 0.39a	29.30 ± 0.81a	35.80 ± 1.49d	32.50 ± 0.31a	4.50 ± 0.13a
Chickpea flour	23.75 ± 0.91a	20.00 ± 0.43b	3.20 ± 0.09c	50.59 ± 1.09c	12.00 ± 0.15b	2.50 ± 0.19b

*Note:* Values followed by the same letter in the same column are not significantly different (*p ≥* 0.05).

### 3.2 Nutritional Components of Biscuits

The results of multiple comparison tests on the main factors (flour type and fat content) for the moisture, protein, fat, total carbohydrate, total dietary fiber, and ash contents of biscuits are presented in Table 3. Multiple‐comparison test results showed that flour type significantly affected (*p* < 0.05) the moisture, protein, fat, total carbohydrate, total dietary fiber, and ash contents of biscuits. No significant effect of fat content was found on the moisture, protein, total carbohydrate, total dietary fiber, and ash contents of the biscuits (*p* ≥ 0.05). The moisture content of chia biscuits was significantly lower than that of other biscuits. Chia and chickpea biscuits contained significantly higher protein than wheat biscuits. A similar increase in the protein content of biscuits was reported by Divyashree et al. (2016). According to Divyashree et al. (2016), buckwheat and chia seed flours caused an increase in the protein content of wheat biscuits from 5.11% to 9.15%. Higher protein content of gluten‐free biscuits is important for children. The fat content of quinoa biscuits was not significantly different from that of chickpea and wheat biscuits. A significantly lower total carbohydrate content was observed in the chia biscuits. The total carbohydrate content of wheat biscuits was not significantly different from that of quinoa and chickpea biscuits. Chia biscuits had the highest total dietary fiber content. Chickpea biscuits had significantly higher total dietary fiber content than quinoa and wheat biscuits. The ash content of chickpea biscuits was significantly higher than that of wheat biscuits. The data of chia biscuits is linked with the result of the study reported by Tüter et al. (2025) for chia seed enhanced gluten‐free biscuits. Tüter et al. (2025) found that chia seed caused increases in protein, fat, total dietary fiber, and ash, while a decrease in carbohydrate content of gluten‐free biscuits. Mesías et al. (2016) reported that protein, lipid and total dietary fiber content increased, while carbohydrate content decreased as chia flour percentage increased from 0% to 20% in wheat biscuit. Allouch Tounsi et al. (2025) presented that chickpea flour improved protein and fiber content of gluten‐free biscuits made from maize and rice flours. Semi‐fat gluten‐free biscuits are suitable for obese individuals due to their lower fat content.

**TABLE 3:** Multiple comparison test results of main factors (flour type and fat content) for nutritional contents of biscuits.

	Samples	Moisture (%)	Protein (%)	Fat (%)	Total carbohydrate (%)	Total dietary fiber (%)	Ash (%)	Energy (kcal/100 g)	P (mg/kg)	Ca (mg/kg)	Mg (mg/kg)	K (mg/kg)
Flour type	Wheat	10.30b	8.40b	16.94b	64.04a	2.02c	0.32b	442.24b	561.71c	280.18c	155.64c	179.49b
	Quinoa	8.05c	10.00b	19.70ab	61.73a	3.99c	0.52ab	437.75b	2455.95b	430.98c	1231.95b	2192.96a
	Chia	6.08d	14.04a	33.76a	45.53b	17.52a	0.59a	542.09a	4818.62a	4468.85a	2265.58a	2616.87a
	Chickpea	13.60a	12.96a	17.81ab	55.10ab	6.47b	0.53ab	432.51c	1991.47b	1648.04b	983.20b	2742.36a
Fat content	Semi‐fat	9.63a	11.85a	17.89b	59.69a	7.91a	0.56a	447.16b	2585.74a	1785.98a	1217.26a	2098.83a
	Full‐fat	8.83a	10.85a	26.19a	53.48a	7.10a	0.42a	493.05a	2328.14a	1628.05a	1100.93a	1767.01a

*Note:* Values followed by the same letter in the same column are not significantly different (*p ≥* 0.05).

Abbreviations: Ca, calcium; K, potassium; Mg, magnesium; P, phosphorus.

### 3.3 Energy Value of Biscuits

The results of multiple‐comparison tests for the main factors (flour type and fat content) on biscuit energy values are presented in Table 3. Multiple‐comparison test results showed that flour type and fat content significantly affected the energy value of the biscuits. Chia biscuits had the highest energy value due to their high‐fat content. Because fat gives higher energy than protein and carbohydrate. Chickpea biscuits had the lowest energy values. The energy value of quinoa biscuits was not significantly different from that of wheat biscuits. Semi‐fat biscuits had significantly lower energy value than full‐fat biscuits. Results showed that a semi‐fat chickpea biscuit can be produced for obese individuals due to its less energy than wheat, quinoa, and chia biscuits. Furthermore, it had higher protein than wheat and quinoa biscuits. This property could increase its preference.

### 3.4 Mineral Content of Biscuits

The results of multiple‐comparison tests on the main factors (flour type and fat content) for phosphorus, magnesium, calcium, and potassium content in the biscuits are presented in Table 3. Multiple‐comparison test results showed that flour type significantly affected the phosphorus, magnesium, calcium, and potassium contents of the biscuits (*p* < 0.05). The fat content had no significant effect on the phosphorus, magnesium, calcium, and potassium contents of the biscuits (*p* ≥ 0.05).

The phosphorus content of quinoa, chia, and chickpea biscuits was significantly higher than that of wheat biscuits. The phosphorus contents of quinoa and chickpea biscuits were not significantly different. Among gluten‐free biscuits, the highest phosphorus content was found in chia biscuits. The magnesium content of quinoa, chia, and chickpea biscuits was significantly higher than that of wheat biscuits. The magnesium contents of quinoa and chickpea biscuits were not significantly different. Among gluten‐free biscuits, chia biscuits had the highest magnesium content. The calcium content of chia and chickpea biscuits was significantly higher than that of wheat biscuits. Wheat and quinoa biscuits had not significantly different calcium content. Among gluten‐free biscuits, chia biscuits had the highest calcium content. The potassium content of quinoa, chia, and chickpea biscuits was significantly higher than that of wheat biscuits. The potassium contents of quinoa, chia, and chickpea biscuits were not significantly different. These results are consistent with those reported by Divyashree et al. (2016). Divyashree et al. (2016) reported that buckwheat and chia seed flours caused increases in the potassium and calcium contents of wheat biscuits from 188 to 217, and 18.78 to 74.3 mg/100 g, respectively. Allouch Tounsi et al. (2025) demonstrated that chickpea flour improved the mineral content of gluten‐free biscuits made from maize and rice flours.

Minerals show a wide variety of functions. Phosphorous metabolism is required in biological processes, cellular metabolism and bone mineralization (Itkonen and Lamberg‐Allardt 2023). Magnesium regulates energy metabolism, cardiovascular health, immune‐defense, bone integrity and a physiological well‐being. Magnesium defiency is common worldwide. Low magnesium stature is related with hypertension, diabetes, migraines, osteoporosis, inflammation and depression (Sarić et al. 2025). Potassium has several health benefits. Potassium reduces blood pressure and influences the risk of hypertension, stroke, and coronary heart disease. Potassium improves bone health. Potassium has a protective effect against bone loss. Potassium has an opposing action on calcium excretion. Potassium reduces the risk of kidney stones (Weaver 2013). Changes in bone mineral density, including osteoporosis (affecting about 70% of celiac patients), are related to altered absorption of calcium (Kamycheva et al. 2017). In children, growth retardation and short stature can raise the suspect of an underlying celiac disease. Therefore, their presence in food is essential for health metabolism (Krzywicka et al. 2014). The phosphorus, magnesium, and potassium contents of gluten‐free biscuits, which are important for celiac patients, were significantly higher than those of wheat biscuits. This means quinoa, chia, and chickpea biscuits are more valuable than wheat biscuits in terms of phosphorus, magnesium, and potassium content.

### 3.5 Dimensional Factors of Biscuits

The results of multiple‐comparison tests for the main factors (flour type and fat content) on the diameter, thickness, and spread ratio of biscuits are presented in Table 4. Multiple‐comparison test results showed that flour type and fat content significantly affected the diameter, thickness, and spread ratio of the biscuits (*p* < 0.05). The diameter value of chia and chickpea biscuits was significantly higher than that of wheat biscuits. The diameters of wheat and quinoa biscuits were not significantly different. The highest diameter (67.6 mm diameter) was observed for full‐fat chia biscuits. The fat content of chia biscuits was higher than that of wheat biscuits. Biscuits expanded during baking due to melting of fat in dough. So a higher diameter and spread ratio are obtained (Pareyt et al. 2009).

**TABLE 4:** Multiple comparison test results of main factors (flour type and fat content) for diameter, thickness, and spread ratio values of biscuits.

	Samples	Diameter (mm)	Thickness (mm)	Spread ratio
Flour type	Wheat	62.08c	10.43a	6.04c
	Quinoa	62.48bc	8.95c	7.03a
	Chia	64.38ab	10.00b	6.45b
	Chickpea	64.53a	9.98b	6.49b
Fat content	Semi‐fat	61.76b	10.45a	5.94b
	Full‐fat	65.91a	9.23b	7.06a

*Note:* Values followed by the same letter in the same column are not significantly different (*p ≥* 0.05).

The thickness values of quinoa, chia, and chickpea biscuits were significantly lower than those of wheat biscuits. The thickness values of the chia and chickpea biscuits were not significantly different. The lowest thickness (8.3 mm) was observed for full‐fat quinoa biscuits.

The spread ratio value of quinoa, chia, and chickpea biscuits was significantly higher than that of wheat biscuits. The result could be attributed to their gluten‐free content. According to Pareyt and Delcour (2008), the spread ratio of biscuits decreases linearly with increasing gluten content. Spread ratio values of chia and chickpea biscuits were not significantly different. Similar results were found for chia seed enhanced gluten‐free biscuits reported by Tüter et al. (2025). Tüter et al. (2025) observed that chia seed caused increases in thickness, diameter and spread ratio of gluten‐free biscuits. The highest spread ratio (7.62), which is the quality factor, was observed for full‐fat quinoa biscuits. Full‐fat biscuits had higher spread ratios than semi‐fat biscuits. Because the fat melted during baking, causing them to spread.

### 3.6 Color Values of Biscuits

Color is crucial to the initial acceptability of bakery products, and the extent of browning significantly influences the flavor of the final product (Mundt and Wedzicha 2007). Cookies should present a high‐intensity brown color (Granato and Ellendersen 2009).

Multiple comparison test results for the main factors (flour type and fat content) on the L*, a*, b*, C*, h*, and △E* parameters, as well as the browning index of the biscuits, are presented in Table 5. C* measures the color intensity (Granato and Ellendersen 2009). h* is a degree of color saturation (Pestorić et al. 2019). Multiple comparison test results showed that flour type caused significant changes (*p* < 0.05) in L*, a*, b*, C*, h*, and △E* parameters, and the browning index of biscuits, while fat content caused significant changes (*p* < 0.05) in L*, a*, b*, C*, and △E* parameter and the browning index of biscuits. Wheat biscuits were lighter than gluten‐free biscuits. The L* color value of the chickpea biscuits was significantly higher than that of the quinoa and the chia biscuits. Chia biscuits were darker than quinoa, chickpea, and wheat biscuits due to their black color. A similar case was reported by Divyashree et al. (2016). According to Divyashree et al. (2016), buckwheat and chia seed flours darkened wheat biscuits. The redness of chia biscuits was significantly lower than that of quinoa and chickpea biscuits. The redness of gluten‐free biscuits was significantly higher than that of wheat biscuits. The yellowness of chickpea biscuits was significantly higher than that of quinoa, chia, and wheat biscuits. There was not significant difference in the yellowness of wheat biscuits and quinoa biscuits (*p* ≥ 0.05). Chia biscuits were perceived as having a less intense and saturated color (lower C* and h* values). Chickpea biscuits were perceived as a more saturated color (higher h* values). The total color difference (△E*) between the control biscuit and the gluten‐free biscuits was higher than 3. As perceived by the human eye, all the gluten‐free biscuits were visibly paler than the control biscuits (Cervini et al. 2021). Chickpea biscuits had the highest browning index. Full‐fat biscuits were brighter than semi‐fat biscuits. The redness and yellowness of full‐fat biscuits were lower than those of semi‐fat biscuits. The browning index of full‐fat biscuits was lower than that of semi‐fat biscuits. Previous studies showed a similar trend. Tüter et al. (2025) reported that L*, a*, and b* color values of gluten‐free biscuits decreased with chia seed substituent. L*, a*, and b* color value decreased as the chia flour ratio (0%–20%) increased in wheat biscuits (Mesías et al. 2016).

**TABLE 5:** Multiple comparison test results of main factors (flour type and fat content) for color values, pH, and hardness of biscuits.

	Samples	L*	a*	b*	C*	h*	ΔE*	Browning index	pH	Hardness (g force)
Flour type	Wheat	72.82a	7.66c	28.20b	29.42b	1.28a	—	60.90b	9.33a	8193.30a
	Quinoa	54.67c	11.95a	27.08b	29.60b	1.16b	18.54b	88.28a	7.64b	7855.62a
	Chia	34.92d	8.02b	11.96c	14.39c	0.98c	41.25a	62.95b	7.78b	7047.44a
	Chickpea	63.16b	11.68a	33.90a	35.86a	1.24ab	11.70c	93.59a	7.71b	8165.55a
Fat content	Semi‐fat	55.13b	10.84a	25.67a	27.99a	1.14a	24.26a	81.09a	8.22a	13,271.56a
	Full‐fat	57.66a	8.81b	24.89b	26.66b	1.19a	23.40b	71.77b	7.96a	2359.40b

*Note:* Values followed by the same letter in the same column are not significantly different (*p ≥* 0.05).

### 3.7 pH of Biscuits

The results of multiple‐comparison tests for the main factors (flour type and fat content) on biscuit pH are presented in Table 5. Multiple test results showed that flour type significantly affected biscuit pH (*p* < 0.05). The fat content had no significant effect on the pH value of the biscuits (*p* ≥ 0.05). Wheat biscuits had significantly higher pH than gluten‐free biscuits. This was due to the pH of the raw material used in biscuit production (Laganà et al. 2022). The pH values of full‐fat and semi‐fat wheat biscuits (control) were found to be 9.30 and 9.35, respectively. The pH values of gluten‐free biscuits vary between 7.40 and 7.93. Similar results were found for chia enhanced wheat biscuits reported by Mesías et al. (2016). According to this study, the pH of wheat biscuit (8.3) decreased as chia flour percentage increased from 5% (7.6) to 20% (7.3).

### 3.8 Hardness of Biscuits

The results of multiple‐comparison tests for the main factors (flour type and fat content) on biscuit hardness are presented in Table 5. Multiple test results showed that fat content significantly affected biscuit hardness (*p* < 0.05). There was not a significant effect of flour type on the hardness of biscuits (*p* ≥ 0.05). The hardness of full‐fat biscuits was significantly lower than that of semi‐fat biscuits. Adding fat softens the dough and reduces its viscosity. Fat contributes to an increase in length and to a reduction in the thickness of biscuits, which are then characterized by a friable structure (Maache‐Rezzoug et al. 1998). Similarly, in the study reported by Laguna et al. (2013), biscuits with a low‐fat content (50%) were significantly harder than those with a high‐fat content (100%). The explanation is that when fat content is reduced, the flour particles become more hydrated, making their components more accessible to water. Consequently, the gluten also becomes more hydrated, resulting in a tougher dough that yields harder biscuits (Ghotra et al. 2002).

### 3.9 Phenolic Compounds of Biscuits

Phenolic compounds can inhibit enzymes associated with the development of human diseases and have been used to treat various common human conditions, including hypertension, metabolic disorders, infectious diseases, and neurodegenerative diseases. Phenolic compounds exhibit protective effects against oxidative stress and demonstrate anti‐inflammatory, anticancer, anti‐aging, antibacterial, and antiviral properties (Rahman et al. 2021).

The results of multiple‐comparison tests for the main factors (flour type and fat content) on phenolic compounds in biscuits are presented in Table 6. Multiple comparison test results showed that flour type caused significant changes (*p* < 0.05) in some phenolic compounds (vanillic acid, caffeic acid, *p*‐coumaric acid, rosmarinic acid, naringin) of the biscuits, while flour type had no significant effect on 4‐hydroxybenzoic acid, ferulic acid, and quercetin (*p* ≥ 0.05). The fat content had no significant effect on the phenolic compounds of the biscuits. Chia biscuits contained significantly higher levels of vanillic acid and rosmarinic acid than quinoa, chickpea, and wheat biscuits. Chickpea biscuits had significantly higher caffeic acid than quinoa, chia, and wheat biscuits. Significantly higher *p*‐coumaric acid was found in quinoa and chia biscuits. Quinoa biscuits had significantly higher naringin levels than the chia, chickpea, and wheat biscuits. Gluten‐free biscuits made from white quinoa, chia seed, or chickpea flours may offer some health benefits due to their high levels of important phenolic compounds. Similar results were observed for chia enhanced wheat biscuit by Mesías et al. (2016). In this study, chia flour (5%–20%) increased phenolic compounds content of wheat biscuit. Mesías et al. (2016) reported that chia flour caused increases in *p*‐hydroxybenzoic acid, vanillic acid, *p*‐coumaric acid, and caffeic acid content of wheat biscuit, while ferulic acid decreased as chia flour percentage increased. Allouch Tounsi et al. (2025) demonstrated that chickpea flour improved polyphenol content of gluten‐free biscuits made from maize and rice flours.

**TABLE 6:** Multiple comparison test results of main factors (flour type and fat content) for phenolic compounds (mg/kg) of biscuits.

	Samples	4‐hydroxybenzoic acid	Vanillic acid	Caffeic acid	*p*‐Coumaric acid	Ferulic acid	Rosmarinic acid	Naringin	Quercetin
Flour type	Wheat	0.23a	0.64b	9.91c	0.10b	n.d.	0.75b	0.45b	n.d.
	Quinoa	0.90a	1.61b	115.42b	24.26a	122.17	3.54b	54.44a	3.44a
	Chia	1.00a	8.26a	94.37b	14.79a	n.d.	630.06a	4.81b	n.d.
	Chickpea	0.63a	0.16b	261.19a	0.62b	n.d.	3.33b	1.73b	8.74a
Fat content	Semi‐fat	0.76a	3.50a	150.3a	12.08a	35.60a	194.48a	18.31a	4.94a
	Full‐fat	0.60a	1.83a	90.10a	7.80a	25.48a	124.36a	12.41a	1.15a

*Note:* Values followed by the same letter in the same column are not significantly different (*p ≥* 0.05).

### 3.10 Fatty Acid Composition of Biscuits

GC–MS chromatogram of the biscuit sample is shown in Figure 1b. The results of the multiple‐comparison test for the main factors (flour type and fat content) on the fatty acids of the biscuits are presented in Table 7. Multiple comparison test results showed that flour type caused significant changes (*p* < 0.05) in the myristic, palmitic, stearic, oleic, linoleic, and linolenic acids of the biscuits, but was not effective on caproic, caprylic, lauric, pentadecanoic, palmitoleic, 9,12‐hexadecadienoic, and heneicosanoic acids. The fat content had no significant effect on the fatty acids of the biscuits (*p* ≥ 0.05). Palmitic acid in wheat biscuits was not significantly different from that of chickpea biscuits. Quinoa and chickpea biscuits had significantly higher stearic acid content compared to wheat and chia biscuits. Significantly higher levels of oleic acid were found in wheat and chia biscuits. Quinoa, chia, and chickpea biscuits were significantly higher in linoleic acid than wheat biscuits. Chia biscuits contained significantly higher levels of linolenic acid than wheat biscuits. A similar increase in linolenic acid of biscuits was reported by Divyashree et al. (2016). According to Divyashree et al. (2016), buckwheat and chia seed flours caused increases in oleic acid, linoleic acid, and linolenic acid levels of wheat biscuits from 41.81 to 47.77, 6.53 to 11.53, and 0 to 4.88 mg/100 g, respectively. Linolenic acid has been shown to have protective effects against various types of cancer (Brinkman et al. 2011). Chia biscuits may have a significant impact on human health. Gluten‐free biscuits had a better PUFA ratio than wheat biscuits. Similar results were found for chia‐enhanced wheat biscuits reported by Mesías et al. (2016). Mesías et al. (2016) demonstrated that chia flour (5%–20%) increased polyunsaturated fatty acids content of wheat biscuit. In this study, chia flour caused an increase in linolenic acid, while decreases in oleic acid and linoleic acid content of wheat biscuit.

**TABLE 7:** Multiple comparison test results of main factors (flour type and fat content) for fatty acid composition (%) of biscuits.

	Samples	Caproic acid	Caprylic acid	Lauric acid	Myristic acid	Penta decanoic acid	Palmitic acid	Palmitoleic acid	9,12 hexadeca dienoic acid	Stearic acid	Oleic acid	Linoleic acid	Linolenic acid	Heneicosanoic acid
Flour type	Wheat	0.02a	0.22a	1.85a	2.05a	0.06a	45.54a	0.09a	0.06a	20.10b	23.46a	17.56b	9.11b	0.09a
	Quinoa	0.01a	0.18a	1.41a	1.19b	0.03a	36.32b	0.03a	0.01a	33.57a	0.83b	31.30a	0.12d	0.40a
	Chia	n.d.	0.04a	0.40a	0.51d	0.03a	22.14c	0.02a	0.01a	5.99c	14.55a	23.18ab	39.81a	0.08a
	Chickpea	0.02a	0.22a	2.09a	0.96c	0.15a	40.51ab	0.03a	n.d.	41.01a	2.01b	25.93ab	1.06c	0.17a
Fat content	Semi‐fat	0.02a	0.22a	2.12a	1.54a	0.09a	37.61a	0.06a	0.03	27.42a	11.75a	26.71a	13.55a	0.25a
	Full‐fat	0.01a	0.10a	0.75a	0.80a	0.03a	34.64a	0.02a	n.d.	22.91a	8.18 a	22.27a	11.50a	0.11a

*Note:* Values followed by the same letter in the same column are not significantly different (*p ≥* 0.05).

### 3.11 Sensory Profile of Biscuits

The average sensory acceptance scores for fracture, appearance, flavor, chewiness, and overall acceptability of the biscuits are shown in Figure 1c. Sensory analysis is valuable in determining the acceptability level of different gluten‐free biscuits. Wheat biscuits received the highest score. Gluten‐free biscuits received the desired scores (above 4). Biscuits scoring above 4.00 on the total acceptable assessment were regarded as satisfactory based on sensory evaluation (Tüter et al. 2025). Gluten‐free biscuits made from white quinoa, chia seed, or chickpea flours have good quality in terms of fracture, appearance, flavor, and chewiness. Gluten‐free bakery products available in the market are often poor in flavor. The results of this study showed that gluten‐free biscuits with good flavor can be produced. Allouch Tounsi et al. (2025) reported that legume flours including faba bean, chickpea, and lentil positively affected overall sensory attributes, especially the appearance of gluten‐free biscuits made from maize and rice flours.

In terms of appearance scores, the chia biscuits yield the lowest value, consistent with its L*, a*, and b* color values. The fracture scores of the chia biscuits are consistent with the texture values and yield the lowest value among the biscuits. While full‐fat biscuits received the highest appearance scores, their lightness values were also higher than those of semi‐fat biscuits. Full‐fat biscuits with low hardness values received higher scores for fracture from panelists compared to semi‐fat biscuits.

## 4 Conclusion

Biscuits are a good source of nutrition for celiac patients, as they are in demand by all population groups. In this study, the nutritional components of white quinoa, chia seed, and chickpea flours used in biscuit production differed. The spread ratio of gluten‐free biscuits, which is a quality criterion in biscuits, was significantly higher than that of wheat biscuits. The hardness of gluten‐free biscuits was not significantly different from that of wheat biscuits. The sensory quality of food products is a key factor in decision‐making for celiac patients. All biscuits had the desired sensory scores. White quinoa, chia seed, or chickpea flours in gluten‐free biscuits caused significant increases in protein, minerals, phenolic compounds, and polyunsaturated fatty acids.

This study suggests that gluten‐free biscuits with health‐promoting effects can be made from white quinoa, chia seed, and chickpea flours, which may be beneficial for celiac patients. Children also needs gluten‐free biscuits with high nutritional quality. In gluten‐free biscuits, chia biscuits contained the highest protein, total dietary fiber, phosphorus, magnesium, calcium, 4‐hydroxybenzoic acid, vanillic acid, rosmarinic acid, oleic acid, and linolenic acid, chickpea biscuits contained the highest potassium, caffeic acid, and quercetin, quinoa biscuits contained the highest ferulic acid, *p*‐coumaric acid, naringin, and linoleic acid. The scores of quinoa and chickpea biscuits were close to that of wheat biscuits, but chia biscuits received lower. Chickpea biscuits gives the lowest energy. Hardness of chickpea biscuits is higher. Protein, total dietary fiber, calcium, potassium, caffeic acid, quercetin, oleic acid, and linolenic acid of chickpea biscuits were higher than those of quinoa biscuits. If nutrition, texture, and scores are taken in account, chickpea biscuits could be preferred.

Semi‐fat gluten‐free biscuits contained higher protein, minerals, phenolic compounds, and polyunsaturated fatty acids. Semi‐fat chickpea biscuits could be suitable for obese individuals due to giving low energy. These new functional products will bring new developments in the industry.

## Author Contributions

**Seda Yalcin:** investigation, writing – review and editing, conceptualization. **Azize Atik:** conceptualization, investigation, methodology. **İlker Atik:** conceptualization, investigation, methodology.

## Funding

The authors have nothing to report.

## Disclosure

All authors have read and approved the final version of the manuscript. Seda Yalcin had full access to all of the data in this study and takes complete responsibility for the integrity of the data and the accuracy of the data analysis.

## Ethics Statement

The sensory assessment performed in this study was approved by Afyon Kocatepe University Science and Engineering Scientific Research and Publication Ethics Committee (June 24, 2024—No. 2024/33), in accordance with the Declaration of Helsinki.

## Conflicts of Interest

The authors declare no conflicts of interest.

## Data Availability

The authors confirm that the data supporting the findings of this study are available on request.
